# Mr. Goodwin's Cases of Angina Pectoris

**Published:** 1801-10

**Authors:** W. Goodwin

**Affiliations:** Earlsham, Suffolk


### *To the Editors of the Medical and Phyſical Journal.*

GENTLEMEN,

I Have lately met with two caſes of Angina Pectoris, a diſeaſe which has been ſo ably and accurately deſcribed by Dr. Parry; and as they have been ſucceſsfully treated, I take the liberty of ſending you a ſhort account of their hiſtory and method of cure. The Angina Pectoris is at all times a moſt formidable diſeaſe, and I truſt the following ſtatement will be deemed worthy of inſertion in your very valuable and uſeful Journal.

*Earlsham, Suffolk,*
*September* 5, 1801.

I am, &c.
W. GOODWIN.

---

Mr. ———, a reſpectable farmer, much inclined to corpulency, with a ſhort neck, broad cheſt, and about fifty years of age, was firſt affected when walking haſtily, with an oppreſſion and violent pain in his cheſt, beginning at the lower point of the ſternum, extending up to the chin, and down both arms to the wriſts. On ſtopping, theſe ſymptoms abated, and after ſeveral minutes reſt, or lying down on the ground, the diſtreſsing ſenſations gradually vaniſhed. At firſt, he thought the complaint was accidental, and took little notice of it, being otherwiſe in good health; and when the fits occaſionally recurred, he often tried to diſſipate them by walking farther, but was always compelled to ſtop, to prevent immediate ſuffocation. In a few weeks the diſeaſe increaſed to ſuch an alarming height that he could ſcarcely walk in the *open air* without the oppreſſion and pain returning; but, what is curious, he could take ſufficient exerciſe *in the houſe* without feeling any attack; neither did the fits return whilſt in bed; however, violent agitation, anger, or conſiderable exertion, would bring them on at any time. At the commencement of this diſeaſe he was ſeldom attacked on horſeback, but as the complaint gained ground, he could only ride the ſloweſt pace without the ſpaſms returning.

He had been ſome weeks under the care of Mr. Revett of Debenham, a very reſpectable practitioner, who had tried bleeding, bliſters to the ſternum, and the warm bath, and had alſo ordered him antiſpaſmodics and opiates, the laſt of which always relieved, although they did not prevent the paroxyſms, yet were found ſo neceſſary as to be conſtantly carried in his pockets. Independent of the complaints I have deſcribed, I

found found my patient healthy and *hearty*, with the means and incli-nation for high living and indulging his appetite.

On the idea that the difeafe was gouty, guaiacum, fudorifics, volatiles, and opiates were prefcribed; a blifter was applied to the fternum, and kept open for fome time; he wore a flannel fhirt, and ftrict attention was paid to his diet: but thefe not producing the defired effects, he applied at different times to two eminent phyficians, one of whom treated the difeafe as dyfpepfia, and the other as gout; unfortunately he experienced no relief from the prefcriptions of either. The cafe grew worfe, and he could with difficulty attend to any bufinefs re-quiring the leaft exercife. In this ftate I faw him a fecond time, fome months after my firft vifit; finding how much had been done to no good purpofe, and learning that his firft attack happened foon after leaving a *very hot and crowded theatre, and exposing his chest suddenly to the cold air*, I propofed applying pieces of callico to the fternum, wetted with the following fo-lution of antimon. tartarizatum, feveral times a day.

℞. Antim. tartar. ʒj. Sp. vin. camph. ℥fs. Aqu. fervent. ℔j. M.

The ftimulus from this application produced an uncommon and violent eruption on the fkin in a fhort time, having the peculiar malignant appearance of carbuncles, itching and fmart-ing exceffively, many of which fuppurated, while hundreds were continually rifing up, fome as large as peas, others as fmall as pin's heads. As foon as the eruption appeared my patient experienced confiderable relief from the fpafmodic affections, and he went on gradually recovering, after conti-nuing the above application two or three times a day for a month.

The fecond inftance occurred in a clergyman fixty years of age, tall, ftout, and athletic; in other refpects in good health. He was attacked with violent pain and oppreffion in the cheft beginning at the fternum, and affecting both the lower arms, and no other parts. The fits were very fevere, remaining a long time, and returning fo frequently that his death was ex-pected at every paroxyfm, and which the patient always thought he warded off by taking brandy. As this gentleman was a patient of Mr. Revett, he applied the following ftrong embro-cation to the fternum, ℞. Ant. tart. ʒj. Linim. fapon. ℥fs. Aqu. fervent. ℥iij. M. in the manner above defcribed, with the like confequence as to the eruption, and with the fame happy effect as in the former cafe, the patient completely reco-vering. In both thefe cafes the arterial fyftem was not mate-rially affected, even during the fits of pain and oppreffion, al-though the patients fuffered little fhort of fuffocation. Neither of the gentlemen took any medicine, or uſed any other remedy whilſt applying the tartarized antimony; and I cannot help flattering myſelf that it may produce ſimilar benefit in other caſes of Angina Pectoris. It is of importance to mention that neither of theſe patients have had any return of the diſeaſe; the firſt has been cured ſix months, and the latter three months.

